# Functional interaction of aortic valve and ascending aorta in patients after valve-sparing procedures

**DOI:** 10.1038/s41598-023-42068-3

**Published:** 2023-09-15

**Authors:** Jan-Christian Reil, Christoph Marquetand, Claudia Busch-Tilge, Maria Ivannikova, Volker Rudolph, Anas Aboud, Stephan Ensminger, Hans-Joachim Schäfers, Ulrich Stierle, Gert-Hinrich Reil

**Affiliations:** 1 Klinik für Allgemeine und Interventionelle Kardiolgie, Herz-und Diabetes-Zentrum Nordrhein-Westphalen, Georgstrasse 11, 32545 Bad Oeynhausen, Germany; 2 Medizinische Klinik II, Kardiologie, Angiologie und Internistische Intensivmedizin, Universitäres Herzzentrum Lübeck, Universitätsklinikum Schleswig-Holstein, Campus Lübeck, Ratzeburger Allee 160, Lübeck, Germany; 3 Klinik für Herzchirurgie, Universitäres Herzzentrum Lübeck, Universitätsklinikum Schleswig-Holstein, Campus Lübeck, Ratzeburger Allee 160, Lübeck, Germany; 4 Klinik für Herz-und Thoraxchirurgie, Universitätsklinkum des Saarlandes, Kirrberger Strasse, 66421 Homburg, Saar Germany; 5 Universitätsklinik für Innere Medizin – Kardiologie, Klinikum Oldenburg, Rahel Strauss Strasse 10, Oldenburg, Germany

**Keywords:** Cardiology, Cardiovascular biology

## Abstract

Pressure recovery (PR) is essential part of the post stenotic fluid mechanics and depends on the ratio of EOA/A_A_, the effective aortic valve orifice area (EOA) and aortic cross-sectional area (A_A_). In patients with advanced ascending aortic aneurysm and mildly diseased aortic valves, the effect of A_A_ on pressure recovery and corresponding functional aortic valve opening area (ELCO) was evaluated before and after valve-sparing surgery (Dacron graft implantation). 66 Patients with ascending aortic aneurysm (mean aortic diameter 57 +/− 10 mm) and aortic valve-sparing surgery (32 reimplantation technique (David), 34 remodeling technique (Yacoub)) were routinely investigated by Doppler echocardiography. Dacron graft with a diameter between 26 and 34 mm were implanted. EOA was significantly declined after surgery (3.4 +/− 0.8 vs. 2.6 +/− 0.9cm^2^; *p* < 0.001). Insertion of Dacron prosthesis resulted in a significant reduction of A_A_ (26.7 +/− 10.2 vs. 6.8 +/− 1.1cm^2^; *p* < 0.001) with increased ratio of EOA/A_A_ (0.14 +/− 0.05 vs. 0.40 +/− 0.1; *p* < 0.001) and pressure recovery index (PRI; 0.24 +/− 0.08 vs. 0.44 +/− 0.06; p < 0.0001). Despite reduction of EOA, ELCO (= EOA corrected for PR) increased from 4.0 +/− 1.1 to 5.0 +/− 3.1cm^2^ (*p* < 0.01) with reduction in transvalvular LV stroke work (1005 +/− 814 to 351 +/− 407 mmHg × ml, *p* < 0.001) after surgery. These effects were significantly better in patients with Yacoub technique than with the David operation. The hemodynamic findings demonstrate a valve-vessel interaction almost entirely caused by a marked reduction in the ascending A_A_ with significant PR gain. The greater hemodynamic benefit of the Yacoub technique due to higher EOA values compared to the David technique was evident and may be of clinical relevance.

## Introduction

With Doppler ultrasound the maximum pressure drop (4Vmax^2^) across the aortic valve at the level of the vena contracta can be calculated, neglecting that kinetic energy is lost distally in the aortic root to turbulent flow, but a smaller portion is converted back to potential energy at the sinutubular junction, referred to as pressure recovery (PR)^1^. However, cardiac catheterization allows assessment of the actual gradient between the left ventricle and the adjacent aorta including pressure recovery based on the pressure readings at both measurement sites^1^. Both techniques therefore often measure different gradients across the valve and thus demonstrate the metrological differences of pure Doppler measurements. The phenomenon of PR is an integral part of the fluid mechanics across the aortic and pulmonary valve, especially in compromised valve function^1–5^. To determine the actual pressure gradient across the aortic valve non-invasively, the Doppler Pmax must be corrected with PR to obtain the true gradient as with catheterization. As early as the 1990s, Voelker et al. demonstrated based on fluid-mechanical experiments^6,7^ that the calculation of PR distal to a stenotic aortic valve depends critically on the ratio of EOA/A_A_, the effective valve orifice area (EOA) and the downstream aortic cross-section area (A_A_) adjacent to the sinutubular junction^6^.

Since the 1990s, aortic root aneurysms have increasingly been treated by valve-sparing procedures with Dacron graft replacement such as root remodeling [Yacoub technique (Y)] or reimplantation of the aortic valve [David technique (D)]^8,9^. Little attention has been paid to the hemodynamic importance of surgical ascending A_A_ reduction and its specific influence on valve hemodynamics. Therefore, our aim was to use the Dacron graft replacement as a model to capture the vascular component of PR formation, making A_A_ the dominant determinant of the Voelker ratio. Consequently, we first examined the hemodynamics of the entire cohort of 66 patients with advanced root and ascending aortic aneurysm and only mild hemodynamic impairments at the aortic valve before and after surgery without considering the different techniques. In a second approach, the influence of reduced A_A_ on the formation of PR and the functional valve orifice area (ELCO) for the two different valve-sparing techniques (D vs. Y) and the small subset of patients with at most moderate aortic valve stenosis was examined to determine the hemodynamic impact and potential clinical relevance for these subgroups.

## Methods

### Patients

Sixty-six Patients with aortic root and ascending aortic aneurysm (mean diameter 58 +/− 11 mm) and aortic valve-sparing surgery (32 David technique, 34 Yacoub technique) were routinely investigated by echocardiography between 2014 and 2020 (Table 1) and retrospectively analyzed. Dacron grafts with a diameter between 26 and 34 mm were implanted. The decision on procedure (David vs. Yacoub) was at the surgeon's discretion, with a larger intraoperatively measured annulus diameter (< 28–30 mm, depending on BSA) determined with a Hegar dilator being an indicator of a David operation and vice versa (personal communication, Prof. em. Hans-Hinrich Sievers, Clinic of Cardiac and Thoracic Vascular Surgery, University of Lübeck, Germany, who performed all operations). Most patients showed sinus rhythm (n = 63), and a normal LV ejection fraction (EF 56 +  − 6%); 44 patients had mild aortic regurgitation (AR I°) according to ASE guidelines^10^ and 15 patients mild to moderate aortic stenosis (D = 8; Y = 7), but no other severe valvular heart disease. Both subgroups did not differ in their baseline characteristics. Echocardiography (GE Vivid E9) was performed in these patients immediately before and 4–6 weeks after surgery. Participation was on a voluntary basis and has been performed in accordance with the Declaration of Helsinki. All patients gave their written informed consent for the collection and use of the respective data. The study was approved by the local ethics committee of the University of Lübeck (No. 22-250) and was in accordance with relevant guidelines and regulations of the University of Lübeck.

**Table 1:** Clinical and hemodynamic baseline characteristics of all patients examined, as well as divided into the subgroups [David (D) vs. Yacoub (Y)].

	ALL	David	Yacoub	*p*-value
	n = 66	n = 32	n = 34	
Age	50 +/− 10	51 +/− 11	49 +/− 10	0.55
Sex (m/f)	47/19	24/8	23/11	
Weight (kg)	80 +/− 14	82 +/− 15	78 +/− 13	0.27
Height (cm)	179 +/− 10	181 +/− 8	177 +/− 11	0.10
Electrocardiogram				
Sinus rhythm	63	30	33	0.61
Atrial fibrillation	3	2	1	0.61
Predisposing factors				
Marfan syndrome	10	8	2	0.04
Loeys-Dietz-Syndrome	1	1	0	0.48
Comorbidities				
Hypertension	n = 53	n = 25	n = 28	0.76
Diabetes mellitus	n = 8	n = 4	n = 4	1,0
Coronary artery disease	n = 8	n = 4	n = 4	1,0
Hyperlipidemia	n = 13	n = 8	n = 5	0.36
COPD	n = 2	n = 1	n = 1	1.0
Renal failure	n = 1	n = 1	n = 0	0.48
Aortic regurgitation				
I ° (ASE criteria)	n = 44	n = 21	n = 23	1.0
Aortic stenosis				
1 cm^2^ < EOA < 2cm^2^	n = 15	n = 8	n = 7	0.77
RR Syst/diast	135 +/− 19/81 +/− 14	135 + − 17/82 + − 12	135 + − 20/80 + − 16	0.9/0.55
Echo parameters (pre surgery)				
EF (%)	56 +/− 6	56 +/− 5	55 +/− 6	0.55
LVEDD (mm)	55 +/− 6	54 +/− 6	55 +/− 8	0.52
PW (mm)	13 +/− 2	14 +/− 3	13 +/− 2	0.19
IVS (mm)	13 +/− 2	13 +/− 3	13 +/− 2	0.24
Pmax (mmHg)	12.7 +/− 8.6	16,6 +/− 10.6	9.4*/− 4.1	< 0.01
Flow (ml/s)	349 +/− 83	352 +/− 81	348 +/− 85	0.85
D-aorta (mm)	58 +/− 11	59 +/− 10	58 +/− 10	0.76

Abbreviatins: *BSA* body surface area, *EF* ejection fraction, *LVEDD* left ventricle end-diastolic diameter, *PW* posterior wall, *IVS* interventricular septum, *EOA* effective orifice area, *D-aorta* aortic Diameter, *EOA/A*_*A*_ ratio of effective orifice area and aortic cross-sectional area adjacent to sinotubular junction.

### Theoretical background for pressure recovery and energy loss

**Figure 1:**
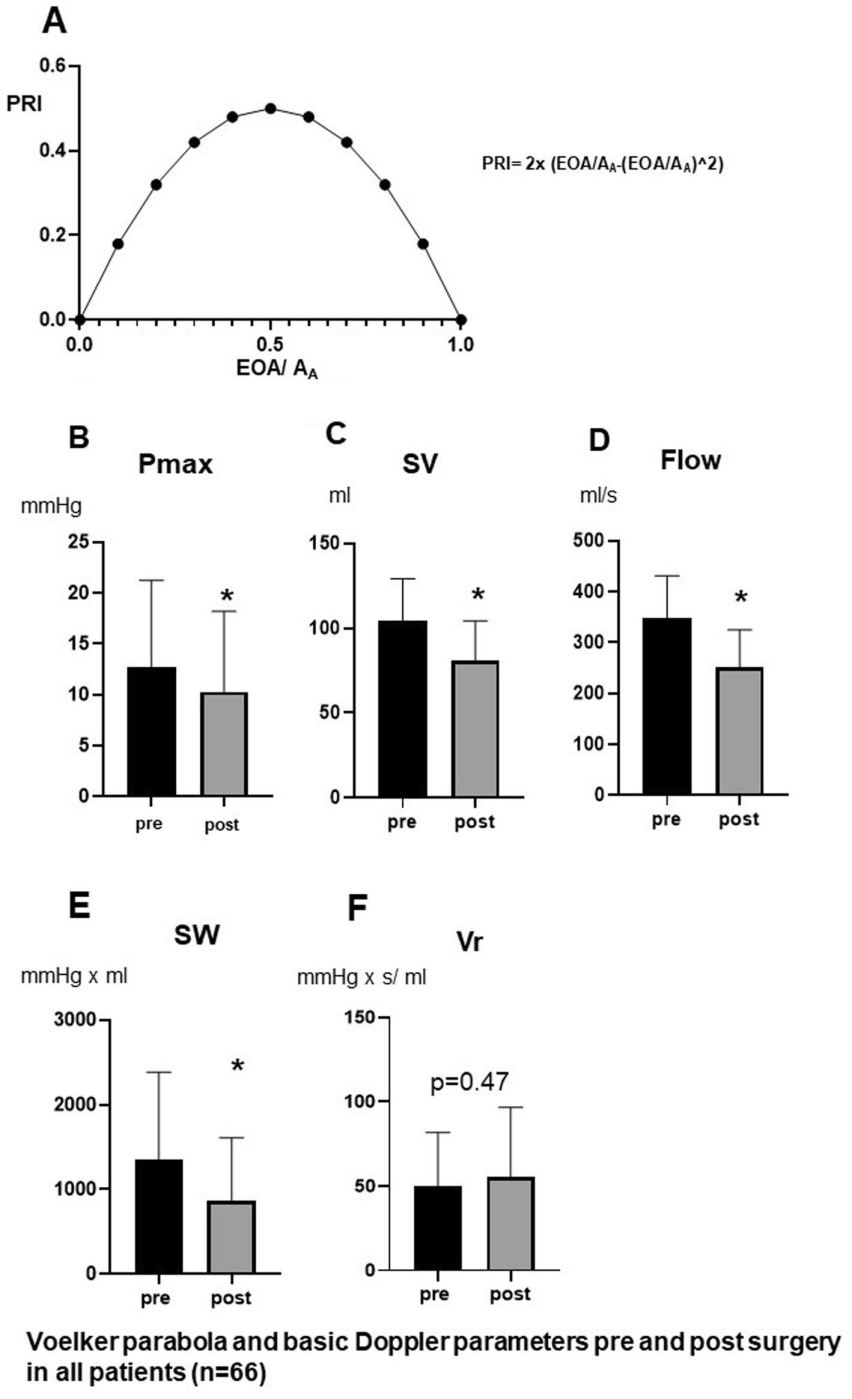
Relation of PRI and EOA/A_A_ describing the „Voelker parabola “ (**A**), Comparison of Pmax (**B**), SV (**C**), flow (**D**), SW (**E**) and Vr (**F**) before and after surgery (n = 66), *p < 0.00001 versus pre surgery.

According to the basic principles of fluid mechanics, conversion of potential energy into kinetic energy occurs downstream to the obstructed aortic valve^2^. Distal to the vena contracta in the aortic root, blood flow decreases and turbulence results in energy loss (EL) by converting kinetic energy into heat^2^. In addition, a smaller part of the kinetic energy is converted back into potential energy, which explains the PR phenomenon adjacent behind the sinutubular junction. The sum of kinetic and potential energy is therefore significantly reduced in the proximal aorta compared to the LVOT and is caused by the energy loss (EL). Cardiac catheterization enables direct measurement of the actual pressure gradient between LVOT and ascending aorta^1,3^. However, the pure Doppler signal (Pmax) cannot capture the PR distal to the measurement point (vena contracta) in the ascending aorta and therefore causes the Doppler gradient to be overestimated compared to invasive catheter measurement. To overcome these Doppler limitations, equations using Doppler-derived parameters were developed to calculate PR and the corresponding “functional valve orifice area”, referred to as energy loss coefficient (ELCO) and means EOA corrected for PR. The problem was solved by developing the Voelker equation (see below) based on the ratio of EOA/A_A_, the effective orifice area (EOA) and the cross-sectional area of the ascending aorta (A_A_)^1^, forming a parabolic function (Fig. 1A).

### Definition of parameters

$${\text{Maximal}}\;{\text{ pressure}}\;{\text{ gradient:}}\; {\text{Pmax}} = {\text{4Vmax}}^{{2}}$$

Pmax could be derived from Doppler measurements, where Vmax is the maximum velocity derived from CW Doppler

$${\text{Energy}}\;{\text{loss}}: {\text{EL}} = {\text{4Vmax}}^{{2}} \times \left( {{1} - {\text{EOA}}/{\text{A}}_{{\text{A}}} } \right)^{{2}} \;\;(11)$$

where EOA is the effective orifice area of the aortic valve when using continuity equation and A_A_ is the cross-sectional area of aorta.

It is calculated by A_A_ = (D/2)^2^ × π (D = diameter of proximal aorta)

$${\text{Pressure }}\;{\text{recovery}}: {\text{PR}} = {\text{4Vmax}}^{{2}} \times {\text{2EOA}}/{\text{A}}_{{\text{A}}} \times \left( {{1} - {\text{EOA}}/{\text{A}}_{{\text{A}}} } \right)\,\,(1)$$

$${\text{Pressure}}\;{\text{recovery}}\;{\text{index}}\left( {{\text{PRI}}} \right) = {\text{relative}}\;{\text{ pressure }}\;{\text{recovery}}$$

$${\text{PRI}} = {\text{PR}}/{\text{Pmax}} = {\text{Pmax}} \times {2}\left( {{\text{EOA}}/{\text{A}}_{{{\text{A}} }} - \left( {{\text{ EOA}}/{\text{A}}_{{\text{A}}} } \right)^{{2}} } \right)/{\text{Pmax}}$$

$$= {\text{PR}}/{\text{Pmax}} = {2}\left( {{\text{EOA}}/{\text{A}}_{{\text{A}}} - \left( {{\text{EOA}}/{\text{A}}_{{\text{A}}} } \right)^{{2}} } \right)\;\left( {{\text{equation }}\;{\text{of}}\;{\text{Voelker}}\left( {6} \right)} \right)$$

Accordingly, Fig. 1A graphically describes the parabolic function between EOA/A_A_ and the relative pressure recovery (PRI) in the ascending aorta according to Voelker et al.^6^ Mathematically the summit of PRI of 50% can be attained by EOA/A_A_ of 0.5. Smaller and higher values of EOA/A_A_ result in lower values of PRI indicated by a parabolic function.

Effective orifice area of aortic valve when using the continuity equation indexed to body surface area (BSA): EOAI.

Energy loss coefficient index (= functional opening area of aortic valve taking PR into account and indexed to BSA):

$${\text{ELCOI}} = {\text{EOA}} \times {\text{A}}_{{\text{A}}} /\left( {{\text{A}}_{{\text{A}}} {-}{\text{EOA}}} \right)/{\text{BSA}}\;\;\left( {{11}} \right)$$

$${\text{Valve resistance}}: {\text{Vr}} = {\text{P}}\,{\text{mean}}/{\text{Q}} \left( {{\text{Q}} = {\text{flow}}\left( {{\text{ml}}/{\text{s}}} \right)} \right)$$

Valve resistance considering PR:

$${\text{Vr}}_{{{\text{PR}}}} = {\text{mean}}\;{\text{EL}}/{\text{Q}}$$

$${\text{mean}}\;{\text{EL}} = {\text{4V}}_{{{\text{mean}}}}^{2} \times \left( {{1} - {\text{EOA}}/{\text{A}}_{{\text{A}}} } \right)^{{2}} \;\;\; \left( {{11}} \right)$$

Transvalvular stroke work:

$${\text{SW}} = {\text{P}}\,{\text{mean}} \times {\text{SV}} \left( {{\text{SV}} = {\text{stroke volume}}} \right)$$

Transvalvular stroke work considering PR:

$${\text{SW}}_{{{\text{PR}}}} = {\text{mean}}\;{\text{EL}} \times {\text{SV}}{.}$$

### Echocardiographic measurements

Transthoracic Doppler echocardiography was carried out with a Vivid 9 system (GE Healthcare, Oslo, Norway). Details of echocardiographic evaluation have been previously described^12^.

### Valvular hemodynamics

Mean and maximal transvalvular pressure gradients (P_mean_/P_max_) were assessed by using the CW-Doppler signal across the aorta. Stroke volume was calculated using the diameter of the LVOT as well as pulsed wave Doppler VTI of LVOT. SV = D^2^/4 × $\pi$ × VTI_LVOT_ where D is the diameter of LVOT. The diameter of the aorta was measured about. 5 mm above the sinutubular junction of the aorta using parasternal long axis view. This localization was recommended by other authors^1,13^, because at this position the blood flow should be laminar again and thus the pressure recovery should be completed. The ventricular ejection time (t) was measured by assessing the time between the R-wave of ECG and the end of the pulsed-waved Doppler LVOT-VTI signal^10^. Flow was calculated by Q = SV/t.

### Statistics

Continuous variables are presented as mean ± standard deviation. Before starting the statistical analysis, a Kolmogorov–Smirnov test for checking normal distribution of the samples was performed. Changes in hemodynamic parameters were analyzed and compared using paired Student’s t-test, if the data were normally distributed. Otherwise, a Wilcoxon matched-pairs signed rank test was used. Changes in hemodynamic parameters between groups were analyzed and compared using unpaired Student’s t-test, if the data were normally distributed. Pearson correlation was calculated in normally distributed parameters. Binary distributed variables of baseline characteristics of different groups were compared using Fisher’s exact test. *p*-values < 0.05 were considered to reflect statistically significant differences. Statistical analyses were performed with GraphPad Prism 8.1.2.

## Results

Table 2 displays the Doppler sonographic and calculated hemodynamic values before and after Dacron graft implantation for the entire cohort. Figure 1B–F summarizes the pure Doppler-derived parameters without considering PR. The maximum pressure Pmax is slightly but not relevantly reduced after the operation (Fig. 1B). SV and flow were significantly diminished after valve-sparing procedures (Fig. 1 C, D). Stroke work (SW) across the valve was significantly lower after surgery (Fig. 1E), while valvular resistance (Vr) remained unchanged (Fig. 1F).

**Table 2:** Echocardiographic parameters of the entire cohort (n = 66), examined pre and post surgery.

	Pre surgery	Post surgery	*p*-value
Pmax (mm Hg)	12.7 +/− 8.6	10.3 +/− 7.9	< 0.05
SV (ml)	104 +/− 25	81 +/− 24	< 0.01
Flow (ml/s)	349 +/− 83	251 +/− 74	< 0.001
EOA (cm^2^)	3.4 +/− 0.8	2.6 +/− 0.9	< 0.001
D-Aorta (mm)	57 +/− 11	29 +/− 2	< 0.001
A_A_ (cm^2^)	26.7 +/− 10.2	6.8 +/− 1.1	< 0.001
EOA/A_A_	0.14 +/− 0.05	0.40 +/− 0.13	< 0.001
PR (mm Hg)	2.9 +/− 2.0	4.3 +/− 2.7	< 0.001
PRI	0.24 +/− 0.08	0.44 +/− 0.06	< 0.001
ELCO (cm^2^)	4.0 +/− 1.1	5.0 +/− 3.1	< 0.05
EL (mm Hg)	9.5 +/− 6.9	4.5 +/− 6.0	< 0.001
SW_PR_ (mm Hg × ml)	1005 +/− 814	351 +/− 407	< 0.001
Vr_PR_ (mm Hg × s/ml)	38 +/− 27	25 +/− 28	< 0.01

Abbreviations: *A*_*A*_ aortic cross-sectional area, *PR* pressure recovery, *PRI* PR index, *EL* energy loss, *ELCO* energy loss coefficient, *SW*_*PR*_*/V*_*PR*_ Stroke volume/valve resistance corrected for PR. Others: see Table 1.

**Figure 2:**
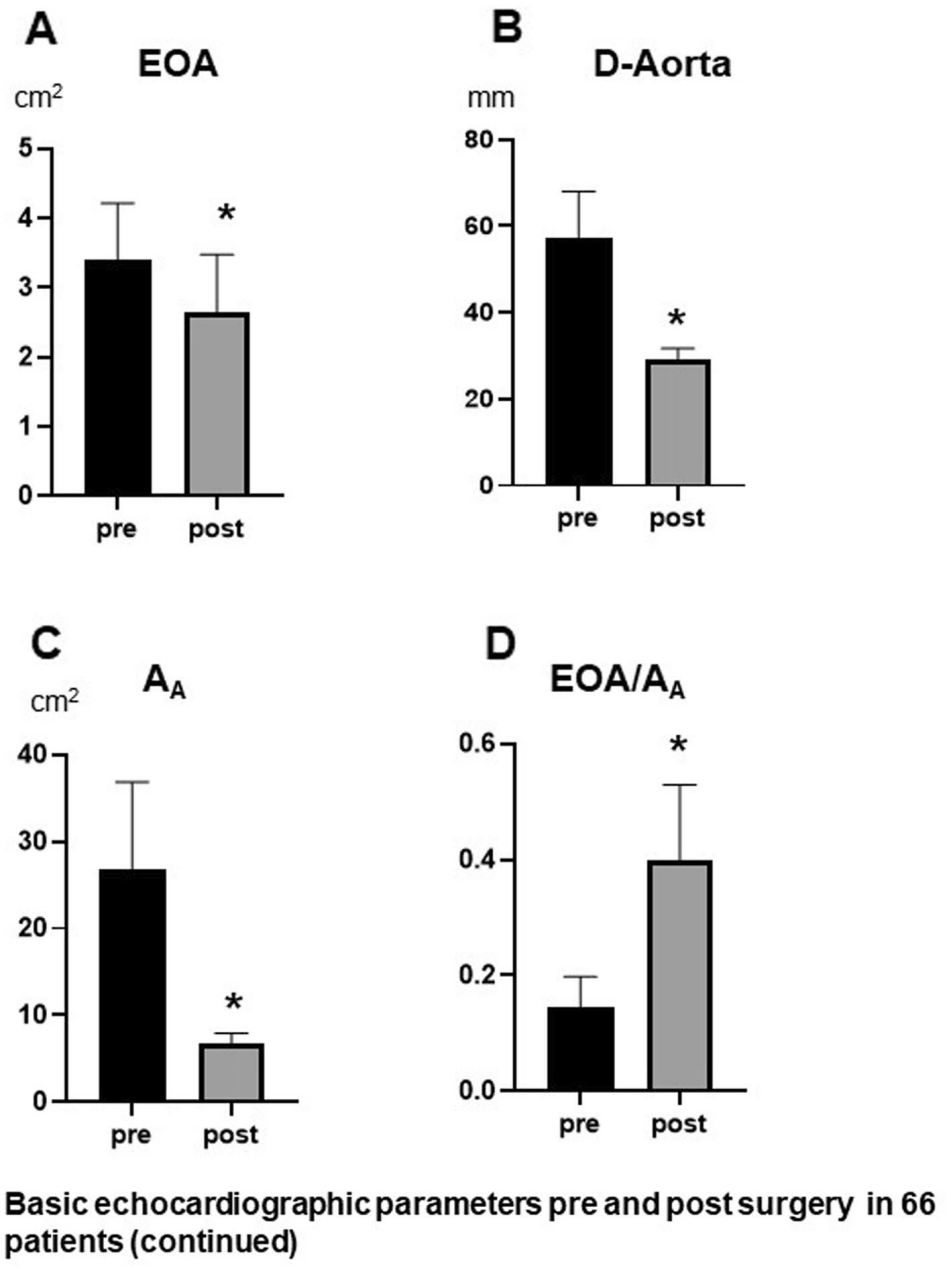
Comparison of EOA (**A**), D-Aorta (**B**), A_A_ (**C**) and EOA/A_A_ (**D**) before and after surgery *p < 0.00001 versus pre surgery (n = 66), *D-Aorta* Diameter of aorta.

**Figure 3:**
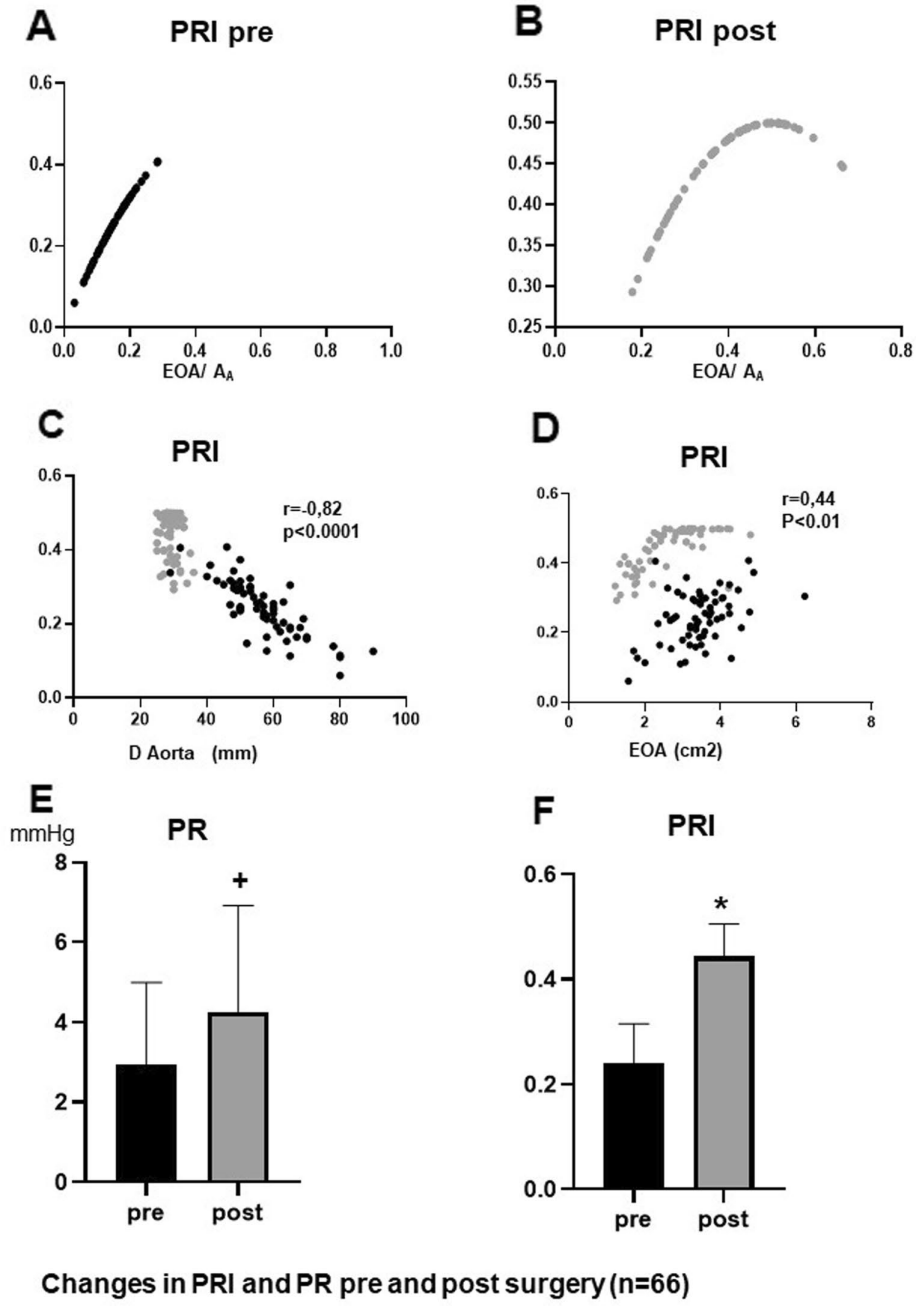
Distribution of the PRI points of the investigated patients on the Voelker parabola before (**A**) and after surgery (**B**). Correlation of PRI and D-Aorta (**C**) as well as PRI and EOA (**D**). Comparison of PR (**E**) and PRI (**F**) before and after surgery (n = 66), *p < 0.00001 versus pre surgery, ^+^*p* < 0.0001 versus pre surgery.

In accordance with these results effective orifice area (EOA) was significantly reduced after surgery (Fig. 2A). Insertion of Dacron graft resulted in a significant reduction of the aortic diameter and aortic cross-sectional area (A_A_) (Fig. 2B,C), so that the ratio EOA/A_A_ increased clearly after surgery (Fig. 2D). This increase is also graphically illustrated in Fig. 3A and B demonstrating the “Voelker parable”—the relationship between PRI and EOA/A_A_. Preoperatively, most patients are in the ascending limb of the parabola up to a maximum PRI of 0.40, while postoperatively the values are shifted to the summit of the curve and beyond, i. e. values between 0.4 and 0.5. This means that reducing (A_A_) resulted in an improved EOA/A_A_ ratio at which optimal pressure recovery can be achieved, as shown in Fig. 3C and D. A good linear correlation could only be demonstrated between the aortic diameter and PRI for all pre and post-surgery points, while there was a weak correlation between the PRI and EOA.

**Figure 4:**
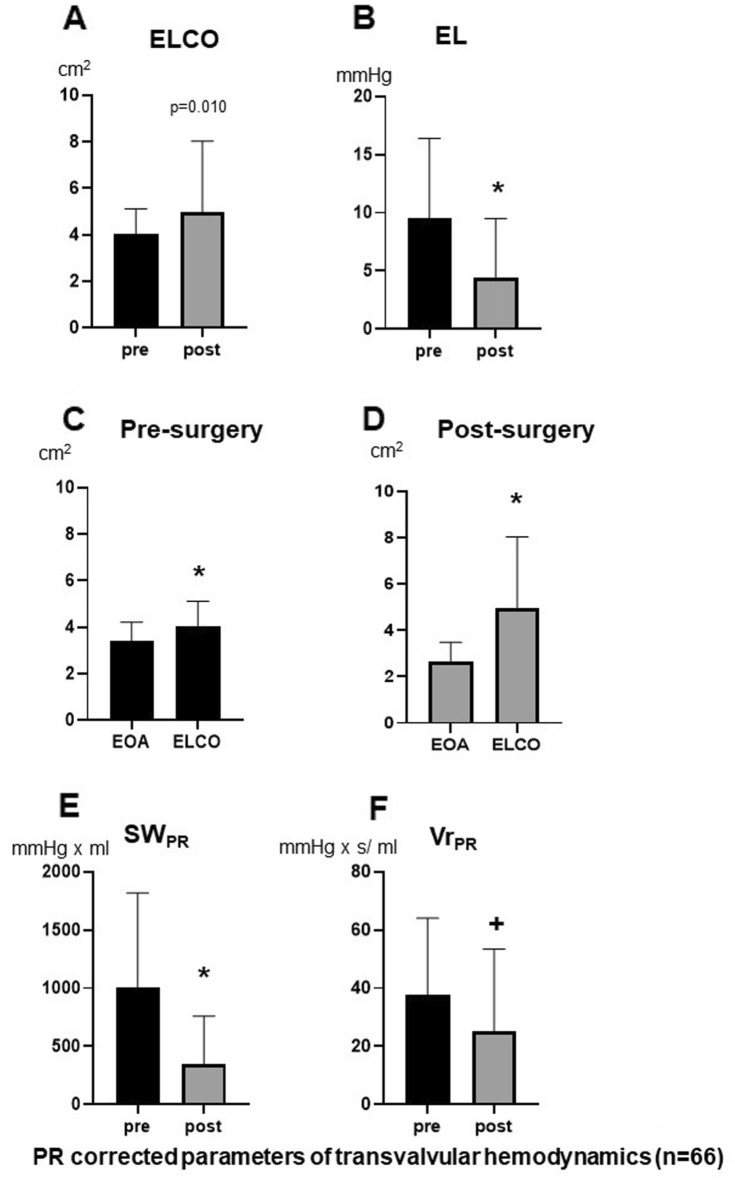
Comparison of ELCO (**A**) and EL (**B**) before and after surgery. Comparison of EOA and ELCO before surgery (**C**) and after surgery (**D**). Comparison of SW_PR_ (**E**) and Vr_PR_ (**F**) before and after surgery (n = 66), *p < 0.00001 versus pre surgery, ^+^*p* < 0.0001 versus pre surgery. SW_EL_/Vr_EL_ = stroke work/valve resistance corrected for energy loss (EL).

Figure 3E and F demonstrate the significant increase of absolute and relative pressure recovery (PRI) caused by the improved EOA/A_A_ ratio. Therefore, ELCO is significantly increased after surgery (Fig. 4A) although EOA was reduced post-operatively (Fig. 2A). Figure 4C andD compare EOA and ELCO before and after surgery. The mean difference between ELCO and EOA increased significantly after surgery (*p* < 0.001). Accordingly, the energy loss across aortic valve was significantly reduced after surgery (Fig. 4B), resulting in significantly reduced stroke work (Fig. 4E) and valvular resistance (Fig. 4F) after surgery.

**Figure 5:**
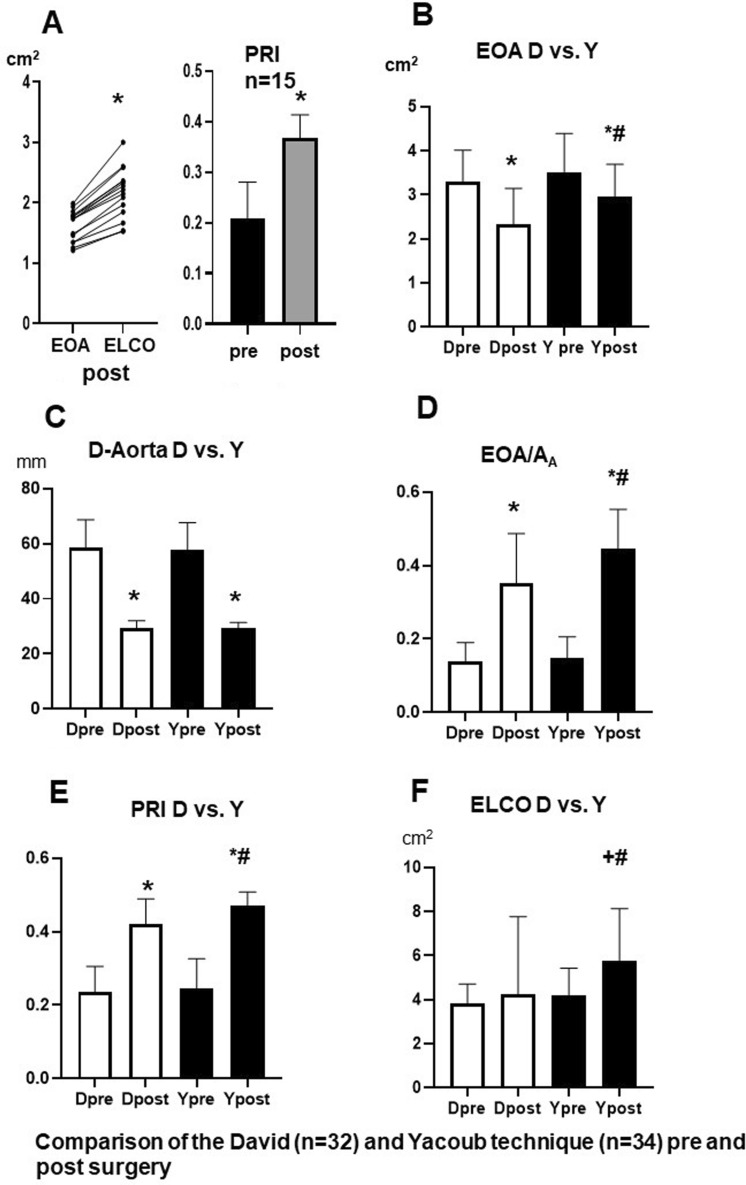
Comparison of EOA and ELCO after surgery (**A**, left side) and comparison of PRI before and after surgery (A, right side) in patients with mild to moderate aortic valve stenosis. (n = 15). *p < 0.00001 versus pre surgery, ^+^*p* < 0.0001 versus pre surgery. Comparison of EOA (**B**), D-Aorta (**C**), EOA/A_A_ (**D**), PRI (**E**) and ELCO (**F**) in patients treated with David (D) or Yacoub (Y) technique before and after surgery. *p < 0.05 versus pre surgery, ^#^*p* < 0.05 post surgery D versus Y.

Fifteen of the investigated patients showed mild to moderate aortic stenosis based on EOA (mean: 1.6 +/− 0.3 cm^2^). If the pressure recovery post-surgery was considered in those patients, the ELCO depicted a significant increase in the valve opening area (mean: 2.2 +  − 0.4 cm^2^). The individual changes are shown in Fig. 5A. It is remarkable that patients with initially moderate stenosis only had minor stenoses and patients with initially minor stenosis consistently had no stenoses when pressure recovery was calculated postoperatively. The corresponding increase of PRI in these patients after surgery is demonstrated in Fig. 5A (right side).

When comparing the remodeling technique (Yacoub) versus the reimplantation technique (David) in our patients (Fig. 5B–D, Table 3), it was striking that the David technique, despite identical preoperative EOA values, resulted in significantly greater gathering of the aortic valve postoperatively with significantly lower EOA in comparison to the Yacoub technique (Fig. 5B). Since both techniques reduced the size of the ascending aorta to the same extent (Fig. 5C), the EOA/A_A_ ratio, however, increased significantly for the Yacoub technique after surgery (Fig. 5D), so that the relative pressure recovery and the functional valve opening area (ELCO) (Fig. 5E, F) were also significantly greater than when using the David technique.

**Table 3:** Echocardiographic parameters of both subgroups [David (D), n = 32; Yacoub (Y), n = 34], examined before and after surgery.

	D pre	Dpost	Ypre	Ypost
Pmax (mm Hg)	16.6 +/− 10.6	13.2 +/− 9.5	9.4 +/− 4.1^#^	7.7 +/− 4.1^#^
Flow (ml/s)	352 +/− 81	245 +/− 70*	348 +/− 85	256 +/− 78*
EL (mm Hg)	12.5 +/− 8.5	6.4 +/− 6.3*	6.9 + 7 − 3.6^#^	2.8 +/− 2.8*^#^
EOA (cm^2^)	3.3 +/− 0.7	2.3 +/− 0.1*	3.5 +/− 0.9	3.0 +/− 0.7*#
D-aorta (mm)	59 +/− 10	29 +/− 3*	58 +/− 10	29 +/− 2*
A_A_	26.5 +/− 11	6.8 +/− 1.3*	26.9 +/− 9.5	6.8 +/− 1.0*
EOA/A_A_	0.12 +/− 0.05	0.35 +/− 0.14*	0.15 +/− 0.6	0.45 +/− 0.11*^#^
PRI	0.24 +/− 0.07	0.42 +/− 0.07*	0.25 +/− 0.08	0.47 +/− 0.04*^#^
ELCO (cm^2^)	3.8 +/− 0.9	4.2 +/− 3.5*	4.2 +/− 1.2	5.8 +/− 2.4*^#^

Abbreviations as in Table 2. * < 0.05 pre- versus post-surgery within a group. ^#^*p* < 0.05 between D and Y.

## Discussion

### The aortic cross-sectional area (A_A_) as the vascular trigger of PR formation

The PR phenomenon has been demonstrated experimentally^14^ and clinically using invasive pressure measurements^4,15^, and can also be precisely calculated by Doppler ultrasound based on Voelker's equation^6^. The area of the proximal ascending aorta just behind of the sinutubular junction is critical for pressure recovery measurements^1,13^. This equation allows EOA and A_A_ to be considered separately as the valvular and vascular components of PR formation, depending on the pathological defect, both determinants have different weights for the calculation of PR. So far, the literature has dealt predominantly with studies on EOA in aortic stenosis as the dominant valvular component of PR formation^1–3^. Aortic aneurysm surgery with Dacron graft replacement as a model made it possible to transfer this weight almost entirely to the vessel side. Our aim was therefore, to emphasize the importance of the vascular side (A_A_) with its special influence on the aortic valve hemodynamics. The hemodynamic findings showed a vessel-valve interaction that leads to a significant PR gain with improved aortic valve functional orifice area (ELCO). The clinical implications of this effect are most evident in the subset of patients with mild to moderate aortic stenosis, while in the subset of patients with different valve-sparing techniques, aortic hemodynamics were slightly better for the Yacoub technique.

### Characteristics of aortic valve-vessel interaction when dominated by A_A_ reduction

The entire cohort of patients after valve-sparing surgery showed a slight reduction in stroke volume, flow and EOA with unchanged Pmax and Vr, which can be explained by the replacement of the aneurysm with the Dacron graft and simultaneous correction of functional aortic regurgitation by gathering of the aortic valve^9^. Flow dependent reduction of EOA may also aggravate this effect^16^. When PR is considered, which is flow independent^6^, two interesting aspects stand out in the data presented.

First, despite the advanced aortic aneurysm, the vessel already showed an unexpected preoperative PR with a significant increase in ELCO and a decrease in EL compared to the pure Doppler-derived measurements of EOA and Pmax. These results demonstrate that PR is even present in the ascending aortic aneurysm with a mean diameter of 5.7 cm (mean A_A_ of 26,7cm^2^) and an EOA of 3.4 cm^2^ resulting in a preoperative ratio of EOA/A_A_ of 0.14, which increased to 0.40 postoperatively. Therefore, our study extends the energy loss principle for A_A_ in a wider range of applicability. So far it has been postulated that no relevant pressure recovery can be achieved with an ascending aortic diameter of more than 3 cm (A_A_ > 7cm^2^) in the case of high-grade aortic valve stenosis^1^. The extension of aortic diameter for PR formation is made possible by a larger EOA (e. g. normal aortic valve), because it shifts the EOA/A_A_ ratio to the right on the “Voelker parabola” towards higher PRI values (Fig. 3A, B). In the past, studies in literature have only focused on the aortic valve opening area as the primary determinant of EOA/A_A_ ratio^3,7,17^. Accordingly, Voelker et al. found no dependence of PR on the valve shape by varying the opening area of the aortic valve, but they did not vary the cross-sectional area of the aorta in their used pulsatile flow model^6^.

Second, our postoperative results clearly demonstrated the importance of reducing the cross-sectional area of the ascending aorta not only to prevent vascular rupture but also to improve aortic valve hemodynamics. These results underscore the functional interaction of the aortic valve and root, initiating valve-vessel “crosstalk”^18^. Even though the aortic valves showed reduced EOA after surgery, ELCO increased significantly while EL, Vr_PR_ and SW_PR_ decreased as expected. This effect was caused by the significantly increased absolute and relative PR, the latter effected by a rightward shift towards the summit of the Völker parabola with a mean PRI value of 0.44 close to the optimum. This shift is mainly triggered by the reduction of aortic cross-sectional area, which is also confirmed by the good correlation between PRI and A_A_, but not for EOA. Accordingly, clinically relevant PR is more common in pediatric patients and young adults, with downstream vessel diameters typically < 3 cm compared to the larger downstream vessels in adults^1,17,19^. Similarly, improved pressure recovery could be shown for the smaller pulmonary artery compared to the aorta^4^.

### The reduction of aortic cross-sectional area and its clinical perspective

The hemodynamic benefit of normal or smaller diameters of the ascending aorta and pulmonary artery increases with the degree of upstream valve obstruction^4,17^. This effect was clearly demonstrated in a subgroup of patients with mild to moderate aortic stenosis after Dacron graft implantation. Due to the smaller Dacron graft diameter, the increased pressure recovery led to a reclassification of the postoperative aortic stenosis from moderate to mild and from mild to no stenosis. In addition to treating the risk of vessel rupture, the improved PR in these patients could be clinically relevant to increase the free interval for possible future valve surgery.

Further considerations appear to be important as to whether a particular surgical technique should be given preference in aneurysm surgery. If one compares the valve- sparing techniques of David and Yacoub, it is striking that in the studied cohort the Yacoub procedure achieves a larger anatomical aortic orifice area, which is reflected in an enlarged Doppler EOA postoperatively compared to the David technique with the same reduction in A_A_. Therefore, Yacoub surgery leads to a better EOA/A_A_ ratio post-operatively probably due to less valve tightening, which results in a better PRI, a higher functional valve orifice area (ELCO) and more unloading of the LV compared to the David technique. The anatomical background of these findings can be explained by clinical and in vitro echocardiographic studies showing better root compliance and almost physiological cusp movement when the Yacoub technique was used^20,21^. From the early postoperative results it could be concluded that patients treated with the Yacoub technique may have improved hemodynamic and possibly clinical advantages over those treated with the David technique, but this needs to be confirmed by long-term hemodynamic and clinical studies.

### Limitations

The present study was retrospective, observational and unicentric and focused only on the hemodynamics of the patient valves examined and therefore could not make any significant statement about the clinical relevance of the various groups. Our main goal was to explain the principle of functional valve-vessel interaction emphasizing the importance of the vascular side through the reduced A_A_. The presented study lacks any invasive data, all parameters were calculated by Doppler echocardiographically-derived measurements. Nevertheless these parameters parameters were invasively validated and clinically checked in literature^1,2,6,17,22^.

## Conclusion

In patients with advanced root and adjacent ascending aortic aneurysm the influence of reduced aortic cross-sectional area on improved pressure recovery, larger corresponding functional aortic valve orifice area (ELCO), and more LV unloading was demonstrated after implantation of the Dacron graft. These results demonstrate a functional interaction of the aortic valve and ascending aorta that initiates valve-vascular “crosstalk” that is primarily determined from the vascular side. Postoperatively, the Yacoub technique showed improved valve hemodynamics compared to the David technique and patients with aortic stenosis were reclassified, which may be of clinical relevance.

## Data Availability

The datasets used and/or analysed during the current study available from the corresponding author on reasonable request.

## Abbreviations

A_A_: Cross-sectional area of aorta ascendens adjacent to the sinotubular junction
BSA: Body surface area
D/Y: David/Yacoub operation
E_a_: Effective arterial elastance
EOA: Effective valve orifice area (continuity equation)
EL: Energy loss
ELCO: Energy loss coefficient (= EOA corrected for PR and is also referred to as “functional valve orifice area”)
EF: Ejection fraction
LV: Left ventricle
LVOT: Left ventricular outflow tract
LVSP: Left ventricular systolic peak pressure
PR: Pressure recovery
PRI: Pressure recovery index (relative PR)
SV: Stroke volume
SW: Stroke work
TAPSE: Tricuspid annular plane systolic excursion
Vr: Valve resistance
VTI: Velocity time integral
